# Tools to Tie: Flower Characteristics, VOC Emission Profile, and Glandular Trichomes of Two Mexican *Salvia* Species to Attract Bees

**DOI:** 10.3390/plants9121645

**Published:** 2020-11-25

**Authors:** Claudia Giuliani, Manuela Giovanetti, Daniela Lupi, Marco Palamara Mesiano, Renata Barilli, Roberta Ascrizzi, Guido Flamini, Gelsomina Fico

**Affiliations:** 1 Department of Pharmaceutical Sciences, University of Milan, Via Mangiagalli 25, I-20133 Milan, Italy; claudia.giuliani@unimi.it (C.G.); gelsomina.fico@unimi.it (G.F.); 2 Ghirardi Botanical Garden, Department of Pharmaceutical Sciences, University of Milan, Via Religione 25, I-25088 Toscolano Maderno, Brescia, Italy; renatabarilli@yahoo.it; 3 Centre for Ecology, Evolution and Environmental Changes, Faculdade de Ciências, University of Lisbon, Campo Grande, 1749-016 Lisbon, Portugal; 4 CREA—Research Centre for Agriculture and Environment, Via di Saliceto 80, 40128 Bologna, Italy; 5 Department of Food, Environmental and Nutritional Sciences, University of Milan, Via Celoria 2, I-20133 Milan, Italy; daniela.lupi@unimi.it (D.L.); palamaramesiano.marco@gmail.com (M.P.M.); 6 Department of Pharmacy, University of Pisa, Via Bonanno 6, I-56126 Pisa, Italy; roberta.ascrizzi@gmail.com (R.A.); guido.flamini@farm.unipi.it (G.F.)

**Keywords:** bees, glandular trichomes, *Salvia blepharophylla*, *Salvia greggii*, Lamiaceae, VOCs, pollinators

## Abstract

A plant can combine physical and chemical tools to interact with other organisms. Some are designed for pollinator attraction (i.e., colors and volatile organic compounds-VOCs); others can act to discourage herbivores (i.e., non-glandular trichomes). Few studies fully address available tools in a single species; notwithstanding, this information can be pivotal in understanding new interactions out of the home range. We characterized flower traits, emission profiles of constitutive compounds from flowers and leaves, micro-morphology of the glandular trichomes, and listed flower visitors of two Mexican bird-pollinated *Salvia* species (*S. blepharophylla* and *S. greggii*), growing in an Italian botanical garden. Flowers were highly variable in their morphometric characteristics. In both species, four trichome morphotypes with similar histochemistry and distribution were documented for leaves and flowers except the calyx abaxial side. The vegetative emission profiles were qualitatively more complex than the floral ones; however, common compounds occurring in high relative percentages were β-caryophyllene and germacrene D. Floral bouquets were dominated by limonene and β-pinene in *S. greggii* and by 1,8-cineole in *S. blepharophylla*. Two potential (non-bird) pollinators were especially abundant: small bees belonging to the genus *Lasioglossum* and large bees belonging to the species *Xylocopa violacea*. Our study highlights the plasticity of these plants, as well as tools that can be conveniently used to establish novel interactions.

## 1. Introduction

In the course of evolution, plants have developed different strategies to attract or repel other living organisms. As attractants, the synthesis of colored substances and the production of volatile organic compounds (VOCs) by glandular trichomes are among the most investigated ones [1,2,3,4,5,6]. As deterrents, epidermal structures may act as physical barriers [7], while the emission of volatiles can be a first “warning shout” against predators [3]. A deeper knowledge of how a plant can employ these tools will greatly help in understanding the evolutionary perspective of ecosystem working conditions. However, information is still scattered and often incomplete, even for single well-studied species. Our study arises in this framework. We were interested in sketching the potential of a given plant species in terms of tools applied to attractive/deterrent performances and their plasticity in actual plant–animal interactions. Therefore, we selected two exotic species with an evolutionary path possibly in contrast with the local occurring interactions. The species, growing in a botanical garden in Italy (well-acclimatized since they were planted many years in advance) belong to the genus *Salvia* (Lamiaceae): *Salvia blepharophylla* Brandegee ex Epling (Figure S1) and *Salvia greggii* A.Gray (Figure S2). They are both native to Mexico, with *S. greggii* extending its home range to the southern region of Texas. They are procumbent ornamental plants widely used in horticulture, with distinctive attractive red flowers for bird pollination [8].

*S. blepharophylla* (eyelash-leaved sage) can reach 60 cm in height at full bloom. The leaves have a serrate margin with evident long trichomes along the edges. Flowers are arranged in loose whorls and are red in color with an orange undertone. *S. greggii* (autumn sage) may reach 1.20 m in height, but it is very variable in size and flower color due to the existence of numerous cultivars. In the wild, leaves are typically ovate and glabrous, with an entire margin, and flowers, gathered in racemes, are scarlet red. The leaf glandular *indumentum* has been investigated in *S. blepharophylla* [9], whereas an ultrastructural characterization of the terpene-producing trichomes exists for *S. greggii* [10]. On the contrary, literature data on the characterization of spontaneously emitted VOCs are lacking for both target species. In the native range, both species are referred to as bird pollinated and possess flowers with an active working lever mechanism [8]. However, a single record of the hummingbird *Calipte costae*, observed on white varieties of *S. greggii* cultivated in California [8], has been reported. In the botanical garden, an entomological survey pointed out that these species also attract bees, at lower abundance and variety [11] in comparison to bee-pollinated *Salvia* species. Overall, the genus *Salvia* is well studied for what concerns the pollination strategies of the numerous species: literature data on the pollination ecology in relation to floral morphology are rich ([12] and literature therein), even though there are no specific studies or inferences correlating such interactions with secondary metabolite production.

The current study provides a comprehensive view of the potential of these species (phytochemical characterization of the VOCs spontaneously emitted from leaves and flowers; presence and distribution of glandular trichomes) and how they may be linked to interactions occurring out of the home range in contrast with previously established pollination syndromes [13,14].

## 2. Results

### 2.1. Floral Traits and Pollinator Monitoring

**Figure 1:**
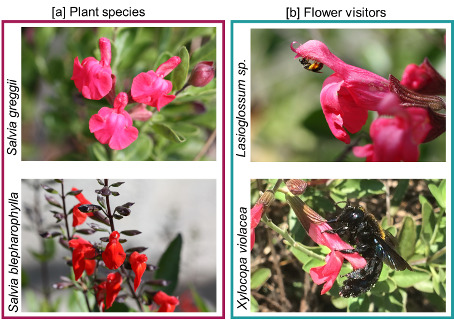
Photographic records of the flowers of [a] *S. blepharophylla* ([a] top) and *S. greggii* ([a] bottom) and of the flower visitors [b] *Lasioglossum* spp. ([b] top) and *Xylocopa violacea* ([b] bottom).

The flowers of the target species are arranged in flowering shoots (Figure 1a). Attraction is enhanced by the dense growth form of the shrubs and by the many simultaneously flowering shoots. The target species present common traits: (i) a hooded upper lip, in which the fertile thecae are hidden; (ii) stigmas protruding out of the upper lips; (iii) a distinct thinner basal part of the corolla tube and a rapidly expanding distal part; (iv) the two lever-like modified stamens typical of the genus; (v) the upper connective arm, bearing a fertile theca, located within the upper lip, while the lower one is long; (vi) the nectary located at the base of the ovary with the nectar accumulating in the thin basal part of the corolla tube.

The two species differ in the following floral characters: (i) the color of the corollas looks red, but their tone is different: it is cold with an orange undertone in *S. blepharophylla*, and warmer with a touch of magenta in *S. greggii*; (ii) the floral proportions differ in the overall flower size, shape, and orientation of the lower lip (slightly reflexed in *S. blepharophylla*, deflexed in *S. greggii*) and among the six examined floral morphological traits (Table 1).

Plant visual display of *S. greggii* and *S. blepharophylla* changed across the blooming period. The total size of *S. greggii* plants ranged from 63 to 120 cm^2^, on average occupying 82.67 ± 16.72 cm^2^ with 32.00 ± 21.10 inflorescences. The average size of *S. blepharophylla* plants was 104.56 ± 11.54 cm^2^ with 43.78 ± 34.39 inflorescences. There were 1–3 available flowers during anthesis on each inflorescence of *S. greggii* and 2–5 flowers on *S. blepharophylla*. Even though, at the inflorescence level, *S. blepharophylla* displayed more flowers than *S. greggii* during most of the observations, the overall display (estimated number of flowers during anthesis on the whole plant during the season) was not significantly different (*t* test, unequal variances assumed = −1.336, df = 9.78, *p* = 0.2119).

**Figure 2:**
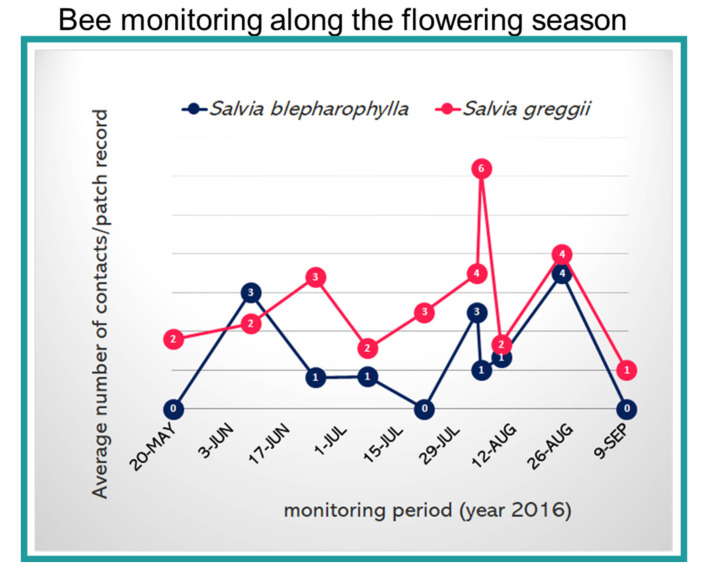
Bee monitoring during the flowering season on the two *Salvia* species. Bee species were summed, with daily average number of contacts during a patch record reported in the graph. In total, we recorded 47 visits on *S. greggii* and 134 on *S. blepharophylla*, distributed across 10 different days and 97 patch records.

Various pollinators, belonging to Hymenoptera, were present in the study area (reported in [11]). However, both sages mainly attracted small *Lasioglossum* spp. and large *Xylocopa violacea* (Figure 1b). Based on the frequency and constancy of their visits, we can certainly conclude that these visits were not random or generic events. The bees repeatedly visited the flowers and collected the resources. We counted 47 visits on *S. greggii* and 134 on *S. blepharophylla*, distributed across 10 different days and 97 patch records. A clear difference between the two species emerged with more visits paid to *S. greggii* than to *S. blepharophylla.* The presence of bees on flowers changed during the season (Figure 2); the trend was not related to each *Salvia* species, but to the time of the year as shown by the overall increase during summer months.

Moreover, the two *Salvia* species were visited by different bees: *S. blepharophylla* only attracted *Lasioglossum* spp. (100% of records), whereas *S. greggii* attracted *Lasioglossum* spp. (84.3% records) plus *X. violacea* (15.7% records). There was a statistical difference in the number of flowers visited for each species: more *S. greggii* flowers were visited (*t* test, unequal variances assumed = 3.390; df = 38.32; *p* = 0.0016). *Lasioglossum* spp. visited both species, mainly interested in the collection of pollen. The same individual may have visited between 1 and 10 flowers during a single foraging bout on *S. greggii*, and between 1 to 3 flowers on *S. blepharophylla*. *X. violacea* visited only *S. greggii* and was interested solely in nectar collection (100% of visits, *n* = 16 records). During our observations, the bee did not contact the pollen, since it accesses it from behind the corolla. However, we cannot state that the bee never visits the plant in a legitimate way, since it may occur at a lower frequency than the opposite behavior. Finally, some individuals were observed visiting the same flower repeatedly.

### 2.2. Glandular Indumenta and Volatile Organic Compounds (VOCs)

**Figure 3:**
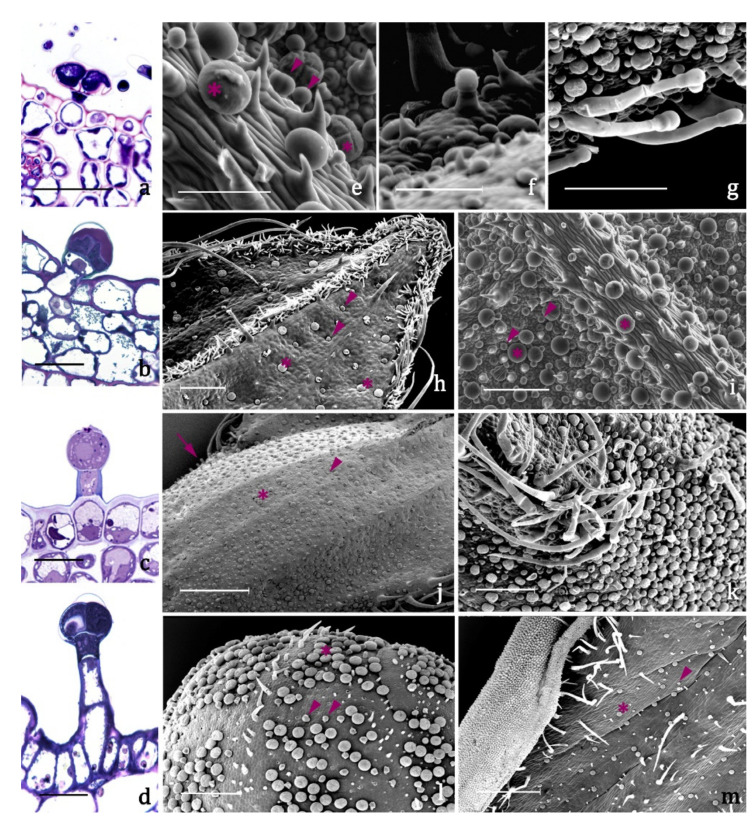
Trichome morphotypes and distribution pattern in *S. blepharophylla* and *S. greggii*. a-d. Light Microscope micrographs. Transverse sections of: (a) type A, peltate trichome. (b) type B, small capitate trichome. (c) type C, medium capitate trichome. (d) type D, long capitate trichome. e-m. Scanning Electron Microscope micrographs. (e). Types A peltate and B short capitate trichomes. (f) Type C medium capitate trichome. (g) Type D long capitate trichome. (h) Leaf abaxial surface of *S. greggii*. (i) Leaf abaxial surface of *S. blepharophylla*. (j) Calyx abaxial surface of *S. blepharophylla*. (k) Calyx abaxial surface of *S. greggii*. (l) Corolla abaxial side of *S. greggii*. (m) Corolla abaxial surface of *S. blepharophylla*. ***Scale bars*** = 25 μm (a–d), 100 μm (e, f, i), 200 μm (g, h, k, l), 500 μm (j, m). **Symbols:** asterisk = type A peltate trichome; arrowhead = type B short capitate trichome; arrow: type C medium capitate trichome.

The glandular *indumenta* of the target species exhibit both peltate and capitate trichomes (Figure 3a–m, Table 2). Four main morphotypes were recognized:

- type A (Figure 3a,e, Table 2), present on leaves and inflorescences of both species (Figure 3h–m), is a typical peltate trichome, constituted by a basal epidermal cell, a neck cell, and by a 4 –cellular glandular head surrounded by a large subcuticular space in which the secretion is stored. The responses to all the lipophilic stains were positive as well as to Ruthenium Red and AlCl3, indicating the presence of terpenes and of major polysaccharide and flavonoid derivatives (Table 3).
- type B (Figure 3b,e, Table 2) is a short capitate trichome, widespread on both the vegetative and the reproductive organs of both examined species (Figure 3h–m). It is constituted by a basal epidermal cell, a neck-stalk cell, and by a glandular head of 2–4 cells surrounded by a wide subcuticular space. Generally, these trichomes present an exclusive polysaccharide secretion released through the intact cuticle (Table 3).
- type C (Figure 3c,f, Table 2) is a medium capitate trichome present only on the calyx of *S. blepharophylla* (Figure 3j). It is made up of one epidermal cell, one stalk cell, one neck cell and a globose head of 1–2 secretory cells surrounded by a storage chamber. The secretion tested positive to all the lipophilic stains, particularly the NADI reagent, indicating that they are exclusive terpene producers (Table 3).
- type D (Figure 3d,g, Table 2) is a long capitate trichome occurring only on the calyx of *S. greggii* (Figure 3k). It is composed by 1–2 epidermal cells, two stalk cells, one neck cell and by a head of 2–4 secretory cells. The secreted material stored in the subcuticular space tested positive only to the lipophilic dyes, indicating the exclusive production of terpenes (Table 3).

Besides glandular trichomes, abundant protective uniseriate trichomes were observed in both species, especially at the edge of the leaves, on the foliar lamina, and along the veins of calyces. In *S. blepharophylla,* they also occurred on the abaxial side of the corolla upper lip. These projections generally point apically toward the top of the organ and are oriented at acute angles to the epidermal surface.

As for VOCs, the flower headspace of *S. blepharophylla* was rich in oxygenated monoterpenes (66.40%) and in sesquiterpene hydrocarbons (22.61%) (Table 4). Among monoterpenes, the most abundant compound was 1,8-cineole (7, 45.68%, Table 4), followed by isobornyl formate (32, 8.56%). The two main sesquiterpene hydrocarbons were β-caryophyllene (50, 6.84%) and germacrene D (64, 5.01%). Among the exclusive compounds accounting for more than 1.00%, we detected isobornyl acetate (37, 1.91%) and trans-cadina-1(6),4-diene (61, 2.65%) (Table 4). In the leaf samples of *S. blepharophylla*, sesquiterpene hydrocarbons accounted for 54.95%, followed by oxygenated monoterpenes (18.83%) and oxygenated sesquiterpenes (13.80%). The most abundant compounds were β-caryophyllene (50, 11.07%), β-bourbonene (43, 10.43%), trans-α-bergamotene (53, 6.89%), and (Z)-β-farnesene (56, 6.82%). Methyl carvacrol (33, 10.68%) was a relevant oxygenated monoterpene, followed by linalool (14, 5.07%). Among the oxygenated sesquiterpenes, the principal one was (Z)-sesquilavandulol (81, 9.63%). Thirty-two exclusive compounds characterized the leaf profile: those occurring in higher relative amounts were linalool (14, 5.07%), trans-α-bergamotene (53, 6.89%), (Z)-β-farnesene (56, 6.82%), and (Z)-sesquilavandulol (81, 9.63%).

In *S. greggii*, the flower volatile profile was mainly rich in terpene hydrocarbons: monoterpenes accounted for 74.78% and sesquiterpenes for 19.08% (Table 4). The main component was limonene (6, 55.20%), followed by β-pinene (3, 14.32%); the two most abundant sesquiterpene hydrocarbons were germacrene D (64, 6.37%) and β-caryophyllene (50, 5.73%). Among the characterizing compounds exceeding 1.00%, myrcene (4, 1.90%) should be mentioned. The leaf samples of *S. greggii* were rich in terpene hydrocarbons: sesquiterpenes reached 42.37%, while monoterpenes accounted for 28.76%. The principal sesquiterpene hydrocarbons were γ-muurolene (62, 10.20%), germacrene D (64, 7.22%), β-gurjunene (52, 6.74%), and β-caryophyllene (50, 5.59%). Among monoterpenes, the most represented compounds were β-pinene (3, 24.96%) and 1,8-cineole (7, 19.56%). Twenty compounds were exclusively present in the leaf profile: the dominant ones were 1,8-cineole (7, 19.56%) and camphor (20, 2.09%) (Table 4).

Although monoterpenes dominated the floral emission profiles of both species, the overall composition appeared diverse. The same was true for the leaf headspace of the two species, where sesquiterpene hydrocarbons prevailed. Among the most abundant compounds emitted by the flowers, only β-pinene (3), methyl carvacrol (33), β-bourbonene (43), β-caryophyllene (50), and germacrene D (64) occurred in both profiles, even if in different relative abundances. In the case of the leaf headspace, α-pinene (1), linalool (14), decanal (31), β-elemene (46) β-caryophyllene (50), germacrene D (64), and (E,E)-α-farnesene (73) were the common compounds. Finally, methyl carvacrol (33), β-bourbonene (43) β-caryophyllene (50), β-copaene (51), and germacrene D (64) were present in the flower and leaf emission profiles of both species and were therefore ubiquitous.

## 3. Discussion

The flowers of *S. greggii* and *S. blepharophylla* are both typically ornithophilous [8], meaning they are characterized by a very long corolla, longer and larger than those of entomophilous species in the same genus, and red in color. The red color should not act as an attractive cue to bees, which are not able to see this color. However, we cannot neglect the possible presence of UV-mediated attraction or the presence of reflectance peaks at wavelengths able to stimulate green or blue bee receptors [8].

According to the evolution towards bird pollination, some morphological modifications evolved to increase the distance between the nectar and the pollen: e.g., elongation of the corolla tube, exposure of the stigma, and reflexion of the lower lip [14]. All these traits co-occur in the two examined species, so that bees are supposedly excluded from resource collection: the elongation of the corolla tube impedes the relatively short proboscis of bees to reach the nectar, and also the pollen, concealed in the upper lip. When compared to existing groups [14] based on flower morphology, the two species belong to group I, the Lanceolata-type, which includes sages with a working lever mechanism. Our morphometric investigation revealed a large variability in terms of different tones of red, overall size, and reciprocal distances between the various floral whorls, even among flowers of the same plant. These differences could also account for the illegitimate visits of *X. violacea*. The absence of differences in the overall floral display indicates other features are involved in the attraction of different pollinators. Differences between the two species emerged regarding the average number of flowers visited during anthesis at any given moment and the tube length, much longer in *S. blepharophylla* than in *S. greggii*. These indicate the flexibility of structures, i.e., flowers may eventually be visited by otherwise unpredicted visitors. In fact, we recorded bees visiting these flowers, even if adopting alternative strategies. *Xylocopa violacea* collected only nectar, its visits being not legitimate [15]: therefore, current data advocate for this species not being a pollinator. However, legitimate visits are possible according to flower traits, and it may still be considered a potential pollinator. Some individuals have been observed repeatedly visiting the same flower, but data are needed to define if this occurs due to leftover nectar or mismatching of attractiveness and actual resource presence (signal-reward correlation) [16]. Harder [17] underlined that bees have restrictions on the tube length they can visit, based on the length of their own glossa. Considering its own length, *X. violacea* was potentially able to legitimately collect nectar from *S. greggii*. However, *X. violacea*, with a proboscis of about 13.00 mm long [18], was equally observed robbing other flowers with corollas within the range of compatibility of its ligula. It is acknowledged that robbing is a constant foraging strategy in this bee species [18] and it is a better explanation for the observed behavior than some incompatibility of bee–flower structures. *Lasioglossum* spp. were rarely recorded looking for nectar, legitimately trying to push the lever mechanism but with evident problems in tripping it due to the small size. However, individuals were very active in collecting pollen: their small size allowed them to hang directly from the anthers. These bees, transferring pollen from one flower to the other, can be considered potential good pollinators of these ornithophilous species.

The glandular *indumentum* is constituted by peltate and capitate trichomes. The peltate trichomes were widespread on all the epidermal surfaces and exhibited a broad four-celled head, as in other *Salvia* species [19]. The peculiar feature of type A trichomes is the exclusive production of polyphenols and flavonoids. These depositions could presumably contribute to the flower visual attractiveness towards some insects, as flavonoids absorb light in the UV range. We recorded two types of capitate trichome on each of the *Salvia* species: type B small capitate occurring on the whole plant epidermis of both species; type C medium capitate and type D long capitate exclusive of the calyx, of *S. blepharophylla* and *S. greggii*, respectively. Type B and C trichomes correspond morphologically to types I and II as described for *S. blepharophylla* leaves [9]. Type B small capitates resulted in exclusive pectic–polysaccharide producers. Inferring that these secretion droplets pass through the intact cuticle, Antunes and Sevinate-Pinto [20] hypothesized that these substances might act as a lubricant to facilitate plant-organ expansion. In type C and D trichomes, the secretory products were exclusively stained by the terpene indicator NADI reagent. The peculiar distribution of types C and D only on calyces is certainly remarkable: they presumably constitute the main sites for the production of the floral volatiles and appear to be mainly located on the abaxial surface, co-occurring with trichomes of types A and B. Therefore, while both leaf and corolla surfaces are comparable in relation to trichome distribution and emission patterns as well, calyces exhibited a peculiar condition: a qualitatively more complex productivity on the abaxial surface due to the presence of three different types of trichomes; an exclusive synthesis of polysaccharides on the adaxial side for the occurrence of only type B. The complex production on the abaxial surface may be primarily related to the potential defensive role of the calyx (as the outermost whorl) in flower buds. Secondarily, at full blooming, the defensive role probably declines while the calyx and its resources enhance attraction.

**Figure 4:**
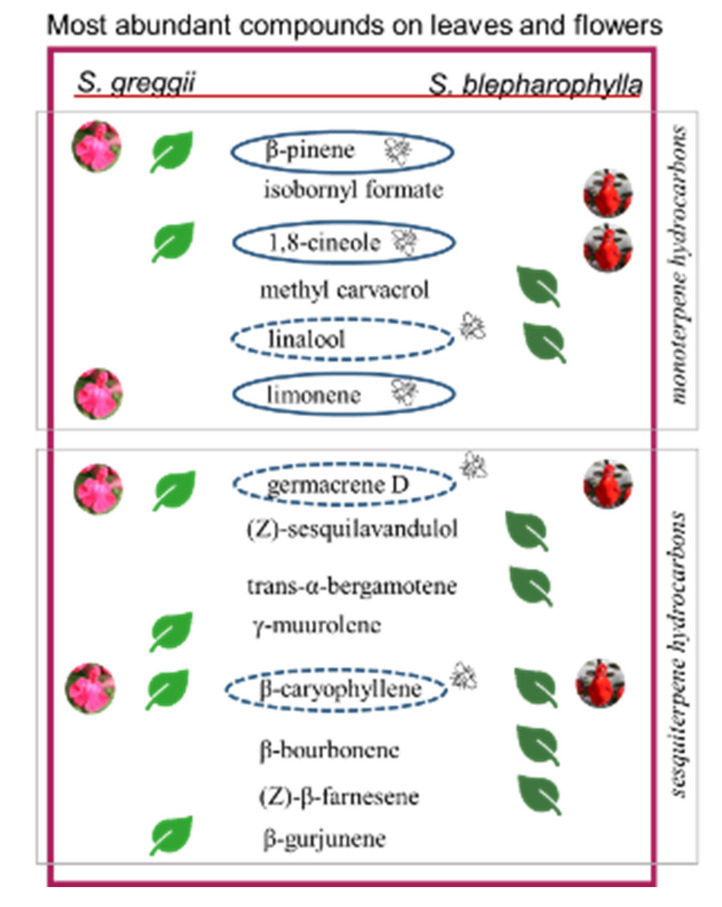
The most abundant compounds found in leaves and/or flowers of the *Salvia* species. Those compounds known from literature to elicit responses by insects are highlighted by continuous (attractive cue) or dotted (deterrent cue) circles.

With regards to the phytochemical data, the flower emission profiles are characterized by the occurrence of one or few exclusive compounds (Figure 4), which may differently affect the interactions between the flowers and the insect pollinators. In *S. greggii,* limonene is massively emitted, followed by β-pinene. In *S. blepharophylla* 1,8-cineole is very abundant, exceeding 45.0%, followed by isobornyl formate. Leaf emission profiles were instead qualitatively more complex. *S. greggii* had 11 compounds with a relative abundance exceeding 2.0%, compared to six in the flower emission profiles. In *S. blepharophylla,* 12 volatiles occurred with relative percentages greater than 2.0% in the leaves, with respect to eight in the flowers. The total profiles of the two species were qualitatively very diverse, excluding the presence of germacrene D, β-caryophyllene and methyl carvacrol that were common to all the analyzed samples. The role of volatiles in bee–plant interactions has been poorly studied due to difficulties in carrying out controlled experiments, and few bee species have been analyzed in relation to plant scents. As evidence of this, the existing information about the chemical cues involved in the attraction of *X. violacea* and *Lasioglossum* spp. to flowers is very rare and refers solely to the latter [21,22]. 

The detection of two bee species in association with *S. greggii* and one with *S. blepharophylla* can be attributed to numerous factors. However, we can also infer that the presence, in their volatile profiles, of compounds emitted by other plants generally visited by these bees might have facilitated the first contact among these native bees and the two *Salvia* species. Certainly, it can be stated that *X. violacea* and *Lasioglossum* spp. found these novel sources interesting, based on the frequency and constancy of their visits. It has been inferred that emissions rich in benzenoids or in linalool (and its oxides) could be an adaptation to a butterfly or to a generalist pollinator [22,23]. Flower attractiveness can be due to a single substance, even if it is more often associated with the total bouquet [24,25]. When the floral bouquet is dominated by a sole volatile in relatively large percentages, the pollination is often bee-mediated [26] (Table 5). 

*S. greggii* and *S. blepharophylla* emitted 1 –2 main compounds in their floral bouquets. Limonene and β-pinene, which are the main volatiles in *S. greggii* flowers, have been demonstrated to be involved in the flower attraction by bumblebees [32] (Table 5) and of Apidae Meliponinae [37]. In addition, 1,8-cineole, which characterizes the flowers of *S. blepharophylla,* appears to have a very important role in the attraction of different bees [26,27,28] (Table 5). According to Granero et al. [28], this compound is also an alarm pheromone for *Bombus terrestris*, which might explain the absence of this pollinator among *S. blepharophylla* visitors. Notwithstanding the intense attraction possibly played by the floral volatiles, we have to keep in mind the mechanical difficulties encountered by bees in handling these flowers, which may have lowered total visitation rates. The physical barriers may also involve the non-glandular and glandular indumenta. The impact of trichome density, length, and orientation on insect behavior and performance has been well documented for herbivores [38,39], while information on the influence on pollinators is scarce or lacking [40].

Finally, volatile biosynthesis is also a defensive response: the production of β-caryophyllene, germacrene D, and linalool can be induced by herbivory [41,42]. Linalool was exclusively emitted by the leaf samples of the studied species, while the other two compounds were also detected in the flower samples. The defensive role of reproductive organs is normally less important in comparison to that of leaves, even if it cannot be completely neglected: a synergistic flower–leaf action may be ascribed to the common emission of deterrent volatiles such as β-caryophyllene or germacrene D, thus ensuring widespread protection throughout the plant. 

In conclusion, our study is the first combining macro- and micromorphological investigations with VOC analyses and direct records of flower visitors. Even if no experimental procedure could be implemented, the study gives a simultaneous prospect of co-occurring circumstances, which allows us to infer newly-established connections between exotic plant species and native bees. Our data highlighted the plastic learning capacity of the local bees, able to bypass physical barriers by adopting peculiar strategies to collect a resource. However, a certain degree of plasticity was certainly also displayed by the plants: notwithstanding their evolutionary path towards bird pollination, they retained some characteristics able to attract insect visitors. The next step would be evaluating the reciprocal benefit, in terms of resource collected for the bees and successful pollination events for the plants and place this in the context of pollination syndromes.

Exhaustively detailing the causes of the different attraction methods of *X. violacea* and the similar attraction by *Lasioglossum* spp. was beyond the scope of this paper. Speculating the substances that may elicit attraction is very difficult in the absence of direct and controlled experiments. Bees are extremely sensitive: for males, slight variability in the relative percentage of the different volatiles in the bouquets of pheromones can strongly impact the attraction potential. The same complexity can be expected by females attracted to flowers, and the complexity may also translate in the same compounds eliciting opposite responses. This was confirmed by the fact that the *S. greggii* bouquet was dominated by limonene and β-pinene: according to literature, these compounds are very attractive to honeybees and bumblebees (Table 5; Figure 4). However, during our surveys, none of these species was detected visiting the plant. Conversely, no information is available on the attraction elicited by 1,8-cineole, which is the dominating substance in *S. blepharophylla*. More multidisciplinary studies are needed in the future to indicate the possible importance of the given compounds and to establish corresponding experiments. 

## 4. Materials and Methods 

### 4.1. Plant Material, Floral Traits, and Pollinator Monitoring

#### 4.1.1. Plant Material

*Salvia blepharophylla* and *Salvia greggii* are cultivated at the Ghirardi Botanical Garden (Toscolano Maderno, BS, Italy) of the Department of Pharmaceutical Sciences of the University of Milan. Plants were identified using the original protologues [43,44], and voucher specimens were deposited in the Herbarium of the Ghirardi Botanical Garden, University of Milan, Italy, under the accession codes UNIMI 0028/15 and 0029/15, respectively. Sampling of leaves and flowers was carried out simultaneously for the micro-morphological and phytochemical studies in June 2016. The macro-morphological investigation and pollinator monitoring were performed throughout summer 2016.

#### 4.1.2. Flower Traits

Thirty randomly selected fully-opened flowers per species were dissected and measured using a digital caliper and a stereomicroscope. Six floral morphological traits per species were selected and measured [44]: (i) calyx length; (ii) flower length; (iii) upper lip length; (iv) lower lip length; (v) length of the corolla tube (measured as the distance between the top of the ovary—where the nectary is typically located—and where the petals separate); (vi) relative length of the upper lip to the corolla tube. The 30 replicates for each parameter were transformed using arcsine square root (arcsin √x) for normalization and then subjected to analysis of variance (ANOVA) to obtain mean values and confidence intervals (α = 0.05). Averages were separated by Tukey’s b post hoc test; *p* < 0.05 was used for the significance of differences between means. The statistical analyses were carried out using the JMP software package (SAS Institute, Cary, NC, USA).

#### 4.1.3. Pollinator Monitoring

We listed flower visitors by randomly recording presence on the flowers on sunny days between 8:00 and 14:00 (local solar hour), through patch records (one observer in a fixed position in front of a *Salvia* plant) lasting 10 min and repeated 2–10 times during the day [45]. Data refer to 10 days, from May to September 2016 fortnightly: 48 patch records on *S. greggii* (totally 480 min) and 49 on *S. blepharophylla* (totally 490 min). Data were normalized according to the number of slots of each day. Plant size (expressed in cm as the projection of the canopy) and number of flowers, as well as weather conditions (descriptive: sunny or cloudy conditions) were recorded at the beginning of each day and of patch observed. Bees were visually identified at least at the genus level. Specimens were also collected for further determination at the species level. Each visit to a single flower was described as (i) resource collected and (ii) number of approached flowers. The entire list of the pollinator fauna in the botanical garden is reported elsewhere [11].

### 4.2. Micro-Morphology of the Glandular Indumentum and Phytochemical Investigation (VOCs)

Leaves, floral buds, and fully open flowers were analyzed by means of scanning electron microscopy and light microscopy to observe: (i) the structure and distribution of the glandular *indumentum* on both vegetative and reproductive organs and (ii) the histochemical nature of the secreted substances. Ten replicates, similar in size, position, and developmental stage, were selected from different individuals for each plant part, in order to verify the consistency of the trichome morphotypes, distribution pattern, and histochemistry.

#### 4.2.1. Scanning Electron Microscopy (SEM) and Light Microscopy (LM)

We combined SEM and LM analyses to describe the trichomes. For SEM samples, fresh leaf, bract, calyx, and corolla samples (5 mm2) were mounted on brass stubs. These samples were viewed in an ambient mode analysis with a QUANTA-200 FEI ESEM.

For LM samples, histochemical tests were performed on hand-cut fresh material to detect the presence of terpenes, lipids, muco-polysaccharides, and phenolics [46,47,48,49,50]. For all the histochemical methods, standard control procedures were carried out simultaneously. All the sections for histochemistry were examined under a light microscope Leitz DM-RB Fluo equipped with a digital camera Nikon DS-L1.

#### 4.2.2. Headspace-Solid Phase Microextraction (HS-SPME) Analyses, Gas Chromatography–Mass Spectrometry (GC-MS) Analyses, and Peak Identification

Three leaves and five complete flowers were cut for each species and inserted into glass vials of suitable volume for the sampling. For HS-SPME sample analysis, Supelco SPME (Solid Phase Micro-Extraction) devices coated with polydimethylsiloxane (PDMS, 100 μm) were used to sample the headspace. SPME sampling was performed using the same new fibre, preconditioned according to the manufacturer instructions, for all the analyses. Sampling was accomplished in an air-conditioned room (22 ± 1°C) to guarantee a stable temperature. After the equilibration time, the fibre was exposed to the headspace for 30 min. Once sampling was finished, the fibre was withdrawn into the needle and transferred to the injection port of the GC–MS system. All the SPME sampling and desorption conditions were identical for all the samples. Furthermore, blanks were performed before each first SPME extraction and randomly repeated during each series. Quantitative comparisons of relative peaks areas were performed between the same chemicals in the different samples. Analyses of leaves were performed in triplicate. Due to their scarce availability, replicates were not considered for flowers.

Gas Chromatography with Electron Impact Mass Spectrometry (GC–EIMS) analyses were performed with a Varian CP-3800 gas-chromatograph equipped with a DB-5 capillary column (30 m × 0.25 mm; coating thickness 0.25 µm) and a Varian Saturn 2000 ion trap mass detector. Injector and transfer line temperatures were kept at 250 °C and 240 °C, respectively; the oven temperature was programmed from 60 °C to 240 °C at 3 °C min^−1^; the carrier gas was helium at 1 mL min^−1^; splitless injection. The mass spectra were compared with those listed in the commercial libraries NIST 14 and ADAMS and in a home-made mass-spectral library, built up from MS literature [51,52] combined with data experimentally obtained from pure substances and commercial essential oils of known composition.

## Figures and Tables

**Table 1:** Floral morphometric variability of the six examined parameters in *Salvia blepharophylla* and *Salvia greggii*: (a) calyx length; (b) flower length; (c) upper lip length; (d) lower lip length; (e) length of the corolla tube (measured as the distance between the top of the ovary—where the nectary is typically located—and where the petals separate); (f) relative length of the upper lip to the corolla tube. The numbers (millimeters) are mean values, with standard error in parentheses.

	a	b	c	d	E	f
***S. blepharophylla***	17.79 ^1^ *(0.13)*	27.21 ^1^ *(0.42)*	10.60 ^1^ *(0.42)*	18.63 ^1^ *(0.38)*	16.61 ^1^ *(0.84)*	0.64 ^1^ *(0.05)*
***S. greggii***	10.50 ^2^ *(0.26)*	24.66 ^2^ *(0.77)*	10.44 ^1^ *(0.39)*	16.40 ^2^ *(0.35)*	14.22 ^2^ *(0.38)*	0.73 ^2^ *(0.01)*

^1,2^ Different superscript numbers indicate significant differences (Tukey’s HSD, *p* ≤ 0.05) for the same parameter (within the same column).

**Table 2:** Distribution patterns of the morphotypes of the glandular *indument*a on leaves and flowers.

	Trichome Morphotype	Leaf		Calyx		Corolla	
		Abaxial	Adaxial	Abaxial	Adaxial	Abaxial	Adaxial
***S. blepharophylla***	A	+	+	+	-	+	+
	B	+	+	+	+	+	+
	C	-	-	+	-	-	-
							
***S. greggii***	A	+	+	+	+	+	+
	B	+	+	+	+	+	+
	D	-	-	+	-	-	-

**Table 3:** Results of the histochemical tests performed on the glandular trichomes. Symbols: (-) absent; (±) scarce, (+) intense, and (++) very intense.

Staining Procedure	Target Compounds	Observed Colour	*S. blepharophylla*			*S. greggii*		
			Type A	Type B	Type C	Type A	Type B	Type D
Nile Red	Neutral lipids	Golden-yellow	++	-	++	++	-	++
Fluoral yellow-088	Total lipids	Yellow to orange	++	-	++	++	-	++
NADI reagent	Terpenes	Violet-blue	++	-	++	++	-	++
FeCl_3_	Polyphenols	Emerald-green	+	-	-	++	-	-
AlCl_3_	Flavonoids	Blue-green	+	-	-	+	-	-
Ruthenium red	Acid polysaccharides	Pinkish to red	+	+	-	+	+	-
Alcian blue	Muco- polysaccharides	Pale-blue	+	+	-	+	+	-

**Table 4:** HS-SPME profiles of leaves and flowers of *S. blepharophylla* and *S. greggii*.

			*Salvia blepharophylla*		*Salvia greggii*	
	l.r.i.^a^	Compounds	Relative Abundance (%)		Relative Abundance (%)	
			Flowers	Leaves	Flowers	Leaves
*1*	941	α-pinene	2.14	-^b^	2.27	3.23
*2*	954	camphene	-	-	-	0.57
*3*	982	β-pinene	2.44	-	14.32	24.96
*4*	993	myrcene	-	-	1.90	-
*5*	1005	α-phellandrene	-	-	0.45	-
*6*	1032	limonene	-	1.66	55.20	-
*7*	1034	1,8-cineole	45.68	-	-	19.56
*8*	1052	(*E*)-β-ocimene	-	3.71	-	-
*9*	1062	γ-terpinene	0.36	0.86	0.22	-
*10*	1070	*cis*-sabinene hydrate	-	-	0.25	0.34
*11*	1076	*trans*-linalool oxide (furanoid)	-	-	-	0.89
*12*	1088	terpinolene	-	0.44	0.42	-
*13*	1090	*cis*-linalool oxide (furanoid)	-	-	-	0.67
*14*	1101	linalool	-	5.07	-	2.11
*15*	1102	nonanal	0.56	-	-	-
*16*	1104	α-thujone	-	0.41	-	-
*17*	1134	*cis*-limonene oxide	-	-	0.20	-
*18*	1140	nopinone	0.75	0.67	-	-
*19*	1141	*trans*-limonene oxide	-	-	3.63	0.08
*20*	1143	camphor	2.95	-	-	2.09
*21*	1156	*iso*borneol	-	-	-	0.17
*22*	1158	sabinaketone	-	0.47	-	-
*23*	1162	*trans*-pinocamphone	-	-	0.15	0.26
*24*	1167	borneol	-	-	-	0.26
*25*	1170	δ-terpineol	-	-	-	0.05
*26*	1178	4-terpineol	1.23	-	0.19	-
*27*	1187	(*Z*)-3-hexenyl-butyrate	0.97	-	-	-
*28*	1192	methyl salicylate	-	-	-	0.14
*29*	1195	γ-terpineol	-	-	0.20	-
*30*	1202	*trans*-dihydro carvone	-	-	0.19	-
*31*	1204	decanal	0.63	1.88	-	0.23
*32*	1232	isobornyl formate	8.56	0.73	-	-
*33*	1241	methyl carvacrol	5.32	10.68	0.61	0.23
*34*	1259	linalool acetate	-	0.80	-	-
*35*	1272	*n*-decanol	-	0.08	-	-
*36*	1283	(*E*)-anethole	-	0.92	-	-
*37*	1285	isobornyl acetate	1.91	-	-	-
*38*	1300	*n*-tridecane	-	0.56	-	-
*39*	1340	δ-elemene	-	0.65	-	-
*40*	1351	α-cubebene	-	-	-	0.05
*41*	1368	cyclosativene	-	-	0.20	0.24
*42*	1376	α-copaene	0.86	-	0.53	2.6
*43*	1384	β-bourbonene	1.20	10.43	0.83	2.74
*44*	1390	β-cubebene	0.28	-	0.23	0.4
*45*	1391	7-*epi*-sesquithujene	-	0.77	-	-
*46*	1392	β-elemene	-	1.05	0.15	0.55
*47*	1400	*n*-tetradecane	-	0.23	-	-
*48*	1403	longifolene	0.41	-	-	0.16
*49*	1409	α-cedrene	0.68	0.28	-	-
*50*	1420	β-caryophyllene	6.84	11.07	5.73	5.59
*51*	1429	β-copaene	0.48	1.33	0.39	0.65
*52*	1432	β-gurjunene	-	-	0.41	6.74
*53*	1438	*trans*-α-bergamotene	-	6.89	-	-
*54*	1439	α-guaiene	-	-	-	0.12
*55*	1441	aromadendrene	-	-	0.35	0.15
*56*	1445	(*Z*)-β-farnesene	-	6.82	-	-
*57*	1455	(*E*)-geranyl acetone	0.41	0.41	-	-
*58*	1456	α-humulene	0.76	2.34	-	0.45
*59*	1461	*allo*aromadendrene	-	1.40	0.16	0.93
*60*	1462	*cis*-muurola-4(14),5-diene	-	0.20	0.27	0.21
*61*	1470	*trans*-cadina-1(6),4-diene	2.65	-	-	-
*62*	1477	γ-muurolene	0.79	-	1.48	10.2
*63*	1480	γ-curcumene	-	0.06	-	-
*64*	1481	germacrene D	5.01	4.22	6.37	7.22
*65*	1490	(*E,Z*)-α-farnesene	-	0.80	-	-
*66*	1491	*trans*-muurola-4(14),5-diene	-	-	0.16	-
*67*	1492	valencene	0.46	-	-	0.51
*68*	1495	bicyclogermacrene	-	2.38	-	-
*69*	1496	γ-amorphene	-	-	-	0.13
*70*	1498	α-muurolene	-	-	0.30	1.08
*71*	1500	*n*-pentadecane	-	0.32	-	-
*72*	1502	γ-patchoulene	0.52	0.10	0.29	-
*73*	1507	(*E,E*)-α-farnesene	-	2.42	0.29	0.82
*74*	1513	*trans*-γ-cadinene	0.98	-	0.70	0.43
*75*	1524	β-sesquiphellandrene	-	1.73	-	-
*76*	1524	δ-cadinene	0.69	-	0.24	0.40
*77*	1549	elemol	-	0.25	-	-
*78*	1565	(*E*)-nerolidol	-	1.29	-	-
*79*	1575	germacrene D-4-ol	-	-	-	0.16
*80*	1576	spathulenol	-	2.16	-	-
*81*	1593	(*Z*)-sesquilavandulol	-	9.63	-	-
*82*	1595	guaiol	-	-	-	0.76
*83*	1600	*n*-hexadecane	-	1.28	-	-
*84*	1606	humulene epoxide II	-	0.14	-	-
*85*	1640	*epi*-α-cadinol	-	-	0.18	-
*86*	1693	juniperol acetate	-	0.33	-	-
*87*	1700	*n*-heptadecane	-	0.08	-	-
		**Monoterpene hydrocarbons**	4.94	6.67	74.78	28.76
		**Oxygenated monoterpenes**	66.40	18.83	5.42	26.71
		**Sesquiterpene hydrocarbons**	22.61	54.95	19.08	42.37
		**Oxygenated sesquiterpenes**	-	13.80	0.18	0.92
		**Phenylpropanoids**	-	0.92	-	-
		**Apocarotenoids**	0.41	0.41	-	-
		**Other non-terpene derivatives**	2.16	4.43	-	0.37
		**Total identified (%)**	**96.52**	**100.00**	**99.46**	**99.13**

*^a^* Linear retention indices on a DB5 column; *^b^* Not detected.

**Table 5:** Floral scent compounds eliciting positive behavioural responses in bees.

Compound	Bee Species	Reference
1,8-Cineole	Euglossini, *Bombus terrestris;* *Bombus vorticosus*	[26,27,28,29]
α-Pinene	Euglossini, *Apis mellifera;* Honeybees	[26,30]
β-Pinene	*Bombus*; Honeybees	[31,32,33]
Limonene	*Bombu*s, Honeybees; *B. terrestris; B. vorticosus*	[29,31]
β-Caryophyllene	*Apis mellifera*;	[29,34]
α-Farnesene		[24]
(*E,E*)-α-Farnesene	*B. terrestris; B. vorticosus; Apis mellifera*	[29,31,35]
Linalool	Colletidae bees; Apidae; *Lasioglossum* spp.	[36]
(*E*)-β-Ocimene	Colletidae bees; Apidae; *Lasioglossum* spp.	[36]

## Supplementary Materials

The following botanical dissection drawings are available online at https://www.mdpi.com/2223-7747/9/12/1645/s1. They were drawn in graphite continuous tone to match the delicacy of the watercolour. We represented the androecium and gynoecium in white inside the corolla, to be shown clearly at first. Watercolour paper Moulin du Roy Canson, graphite Derwent, and watercolour Winsor & Newton were used. Figure S1: Botanical illustration of *Salvia blepharophylla* Brandegee ex Epling; Figure S2: botanical illustration of *Salvia greggii* A.Gray.
