# Supplementary figures and images for: Tools to Tie: Flower Characteristics, VOC Emission Profile, and Glandular Trichomes of Two Mexican Salvia Species to Attract Bees
Source: Plants (Basel). 2020 Nov 25;9(12):1645. doi: 10.3390/plants9121645 (PMC7760984; doi:10.3390/plants9121645)

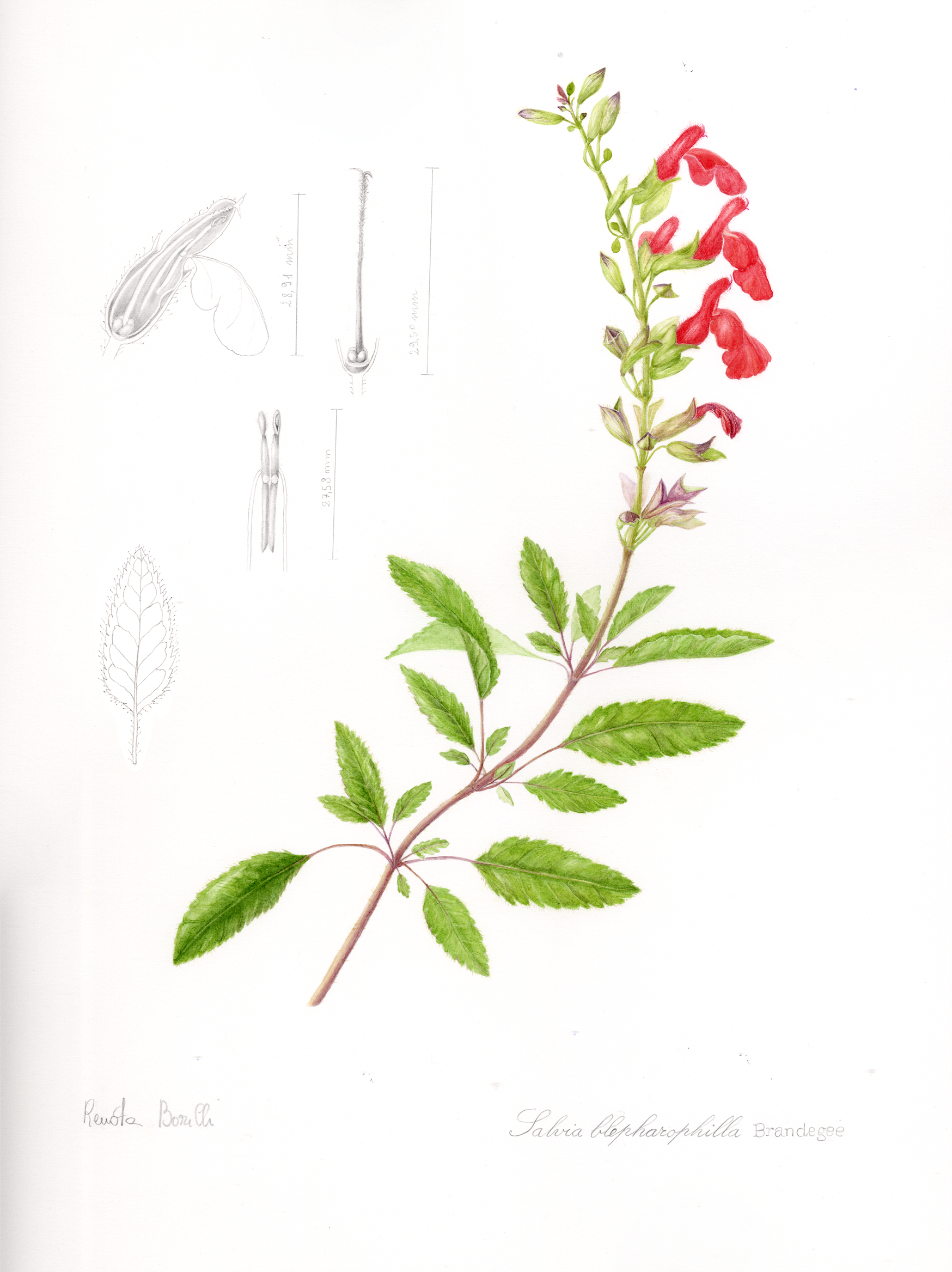

Supplement: Supplementary file 1 [file plants-09-01645-s001.zip › Figure S1_Salvia blepharophylla.tif]

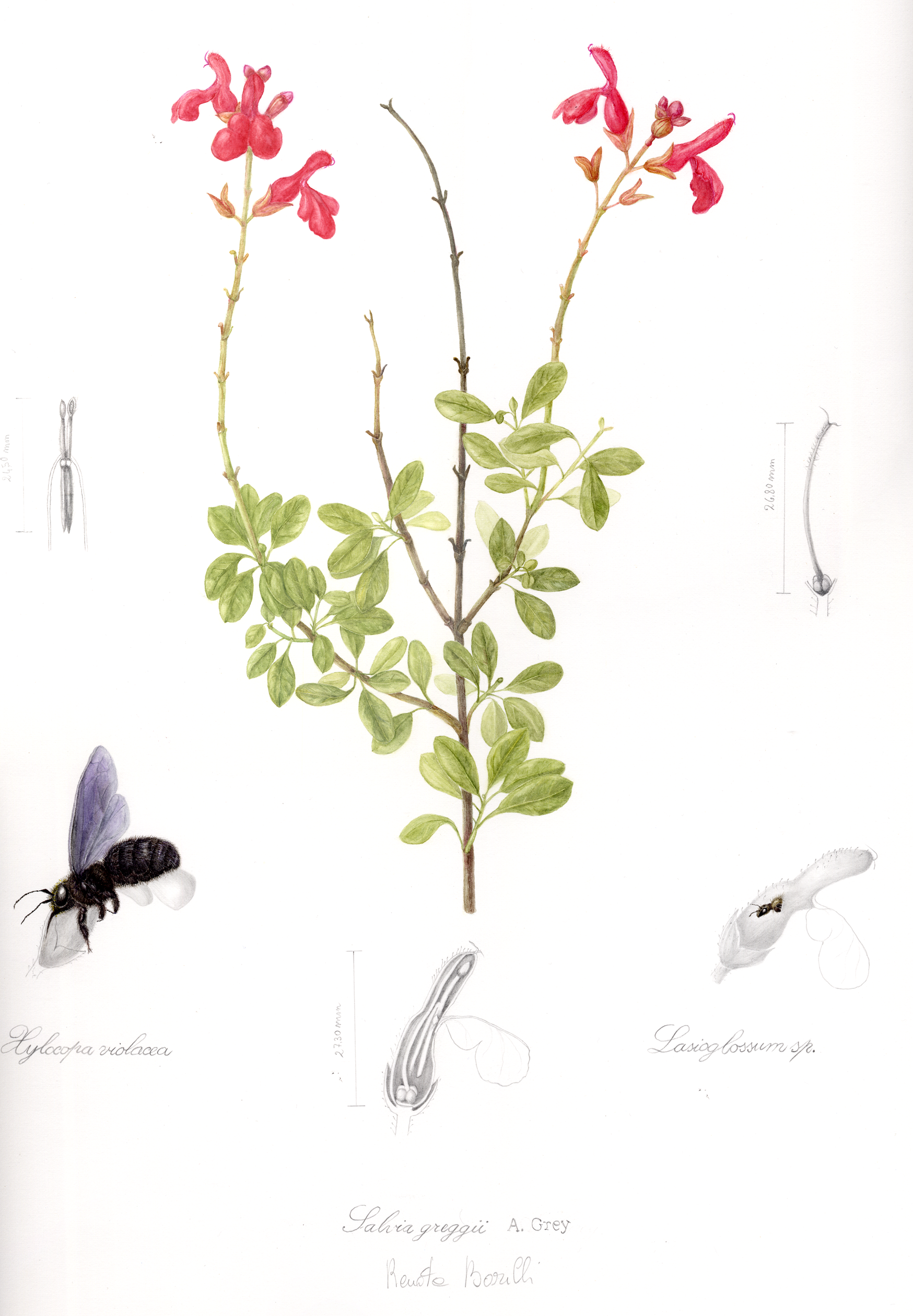

Supplement: Supplementary file 1 [file plants-09-01645-s001.zip › Figure S2_Salvia greggii.tif]
